# Focal adhesion kinase inhibitor BI 853520 inhibits cell proliferation, migration and EMT process through PI3K/AKT/mTOR signaling pathway in ovarian cancer

**DOI:** 10.1007/s12672-021-00425-6

**Published:** 2021-08-30

**Authors:** Hong Li, Yizhi Gao, Chenchen Ren

**Affiliations:** 1 Department of Obstetrics and Gynecology, The Third Affiliated Hospital of Zhengzhou University, Zhengzhou, Henan People’s Republic of China; 2 Department of High School, Wuhan Maple Leaf International School, Wuhan, China

**Keywords:** BI853520, Ovarian cancer, PI3K/AKT/mTOR, EMT

## Abstract

Focal adhesion kinase (FAK) activation has been reported to be associated with cell progression and metastasis in a wide variety of cancer cells. Target treatment by inhibiting FAK has achieved remarkable effects in several cancers, but the effect in ovarian cancer has not been reported. In this study, we determined the role and the underlying molecular mechanism of BI853520, a novel small chemical FAK inhibitor against ovarian cancer. Results show that phosphorylated FAK tyrosine 397 (p-FAK Y397) is highly expressed in ovarian cancer tumor tissues and cell lines (SKOV3 and OVCAR3). BI853520 treatment greatly suppresses cell proliferation, viability, migration, invasion, decreases anchorage-independent growth and motility in vitro. Besides, treatment with BI853520 increases biologic effects following combination with chemotherapy in ovarian cancer cell lines. In addition, BI853520 suppresses EMT in ovarian cancer cell lines. Mechanically, BI853520 treatment downregulates the activation of PI3K/AKT/mTOR signal pathway. Finally, mice model experiments confirm BI853520 treatment dramatically reduces tumor growth in vivo and suppresses the activation of PI3K/AKT/mTOR signal pathway. Taken together, our findings demonstrate that focal adhesion kinase inhibitor BI853520 inhibits cell proliferation, migration, invasion and EMT process through PI3K/AKT/mTOR signaling pathway in ovarian cancer, and BI853520 can offer a preclinical rationale for targeting repression of FAK in ovarian cancer.

## Introduction

Focal adhesion kinase (FAK) is a non-receptor protein tyrosine kinase that forms important focal adhesion sites, which contains various cell surface integrins [1, 2]. Once these integrins recruited by signals from growth factor receptors, FAK undergoes a structural modification, inducing autophosphorylation of the tyrosine 397(Y-397) in N-terminal domain, which indicates the activation of FAK [3, 4].

Phosphorylation of FAK is needed for its kinase activity to induce downstream signals activation such as AKT and mTOR, and trigger various signaling pathways such as PI3K/AKT/mTOR signal pathway, MAPK6/ERK pathway, which was linked to aggressive tumor behaviors [5–7]. PI3K/AKT/mTOR pathway plays a key role in cell proliferation, survival, cell motility, cellular metabolism and cell cycle in various tumor types [8–10]. However, the role of BI853520 in regulating PI3K/AKT/mTOR pathway of ovarian cancer cells has not yet been reported.

Inhibiting FAK phosphorylation prevents uncontrolled ovarian cancer growth caused by FAK hyperactivity. Inhibitors of FAK, such as PF562271, GSK2256098, have been proved to suppress FAK activity and inhibit tumor growth [11–13]. Moreover, several inhibitors of FAK have demonstrated greatly improved the sensitivity of tumor cells to chemotherapy [14, 15]. BI853520 is a specific inhibitor of FAK-Y397 that has shown great antitumor activity [16, 17]. Importantly, BI853520 was proved to have good pharmacokinetics in inhibition mice tumor growth in breast cancer and pleural mesothelioma [16, 18]. However, few studies have evaluated the role and the molecular mechanism of BI853520 in ovarian cancer. This is the first study on the antitumor effects and the underlying mechanism of BI853520 in both vitro and vivo of ovarian cancer.

## Methods

### Clinical samples

Tumor tissues and blood samples were collected from patients who underwent surgery without chemotherapy. This research was approved and supervised by the Hospital Ethics Committee of the Third Affiliated Hospital of Zhengzhou University. All patients signed informed consent.

### Cell lines and reagents

All cell lines were bought from ATCC (Manassas, VA) and cultured in DMEM with10% FBS. Antibodies of FAK, ERK, mTOR, phosphor-Y397 FAK, -mTOR (S2448), -ERK, apoptosis-related genes and EMT marker genes were purchased from Abcam (MA, USA). BI853520 was obtained from Medkoo Biosciences (NC, USA).

### MTT assay

Cells were incubated for 12 h following various doses of BI853520 treatment (0, 0.1, 0.5, 1, 2.5, 5, 10, 25, 50, 75, 100, and 200 μM). Then 20 μL of MTT was added into each well, and 200 μL of DMSO was added after 2 h. The absorbance at 570 nm was detected using a Falcon plate reader.

### Colony formation assay

Cells were counted and cultured in 6-well plates for 24 h, then BI853520 with doses of 2.5–15 μM were added in the wells for 12 h, respectively. The cells were kept for 14 days, and stained using crystal violet.

### Soft agar assay

Firstly, to create the bottom layer of agar growth media, 2 mL of DMEM combined with equal amount of 1% bact-agar solution were put in 6-well plate until solidification. Cells treated with different doses of BI853520 for 12 h were put on the top of the medium-agar layer, and kept in a 37 °C CO_2_ incubator for 4 weeks, and then the colonies cells were counted using Image J.

### Western blot analysis

Protein concentrations were determined as previously described [19]. The protein lysates were separated on 8–10% SDS–polyacrylamide gel, transferred to PVDC membrane, after blocking, the membrane was incubated overnight with primary antibodies. Following incubated 2 h with second antibody, blot bands were scanned using a developer machine with ECL Reagent.

### Cell adhesion assay

Cells were washed, detached and resuspended, and 100 μL cell (2 × 10^5^) were added into 96-well plates, which were coated with the Collagen I solution at 4 °C and then incubated at 37 °C for 20 min. To wash off any non-adherent cells, 100 μL DMEM was added and incubated for 4 h, and 10 μL of MTT was added and incubated for 2 h, and then 100 µL DMSO was added and the absorbance at 570 nm was measured using a spectrophotometer.

### Wound healing assay

Cells were cultured for 24 h, and then the cells were scratched with a 200-μL pipette tip, and washed with serum-free medium for three times. Wound closure was measured both at 0 h and after 24 h. The wound closure percent analyzed using Image J.

### Cell migration assay

Ovarian cancer cells (3 × 10^4^) treated with BI853520 or DMSO in 300 μL serum-free medium were added to the upper chamber, while medium containing 20% FBS was added to the lower chamber and incubated for 24 h. The migrated cells were fixed with methanol and stained with crystal violet. Migrated cells were taken at 10X magnification.

### Cell invasion assay

Ovarian cancer cells (3 × 10^5^) cells treated with 10 μM BI853520 or DMSO were placed in serum-free medium onto upper chamber prepared with Matrigel, while medium containing 20% FBS was added to the bottom chamber for 36 h. The invaded cells were fixed, stained and the photos were taken at 10× magnification.

### Animal model of ovarian cancer

The animal study was supervised by Animal care center of Zhengzhou University. 4 × 10^6^ SKOV3 cells were inoculated into female 8-week-old NSG mice. The mice were divided into following groups: (i) 100 μL of a vehicle control (oral, daily); (ii) 20 mg/kg of BI853520 in DMSO (orally, five times a week); (iii) 2.5 mg/kg of paclitaxel in PBS (intraperitoneal, weekly); and (iv) 20 mg/kg of BI853520 and 2.5 mg/kg of paclitaxel. Therapy was initiated 3 days after tumor injection. Animals were weighed twice a week, and the experiment was ended after six cycles of the treatment. Tumor samples were collected for following research.

### Imunofluorescence staining

The cells or slides were firstly fixed with 4% paraformaldehyde, and incubated with 5% goat serum, 3% BSA, and 0.1% Triton-X100 and incubated with p-FAK, p-AKT or p-mTOR (1:100 dilution, Abcam). Cell nuclei were stained with DAPI. Images were captured under microscope, and the cell fluorescences were measured using Image J.

### Statistical analysis

Data were presented as means ± SD using Student’s *t*-test or one way ANOVO analysis. P < 0.05 was considered different. All data were representative of three independent experiments.

## Results

### Basal phosphorylation of FAK (p-FAK) in ovarian cancer tissues and cell lines

**Fig. 1:**
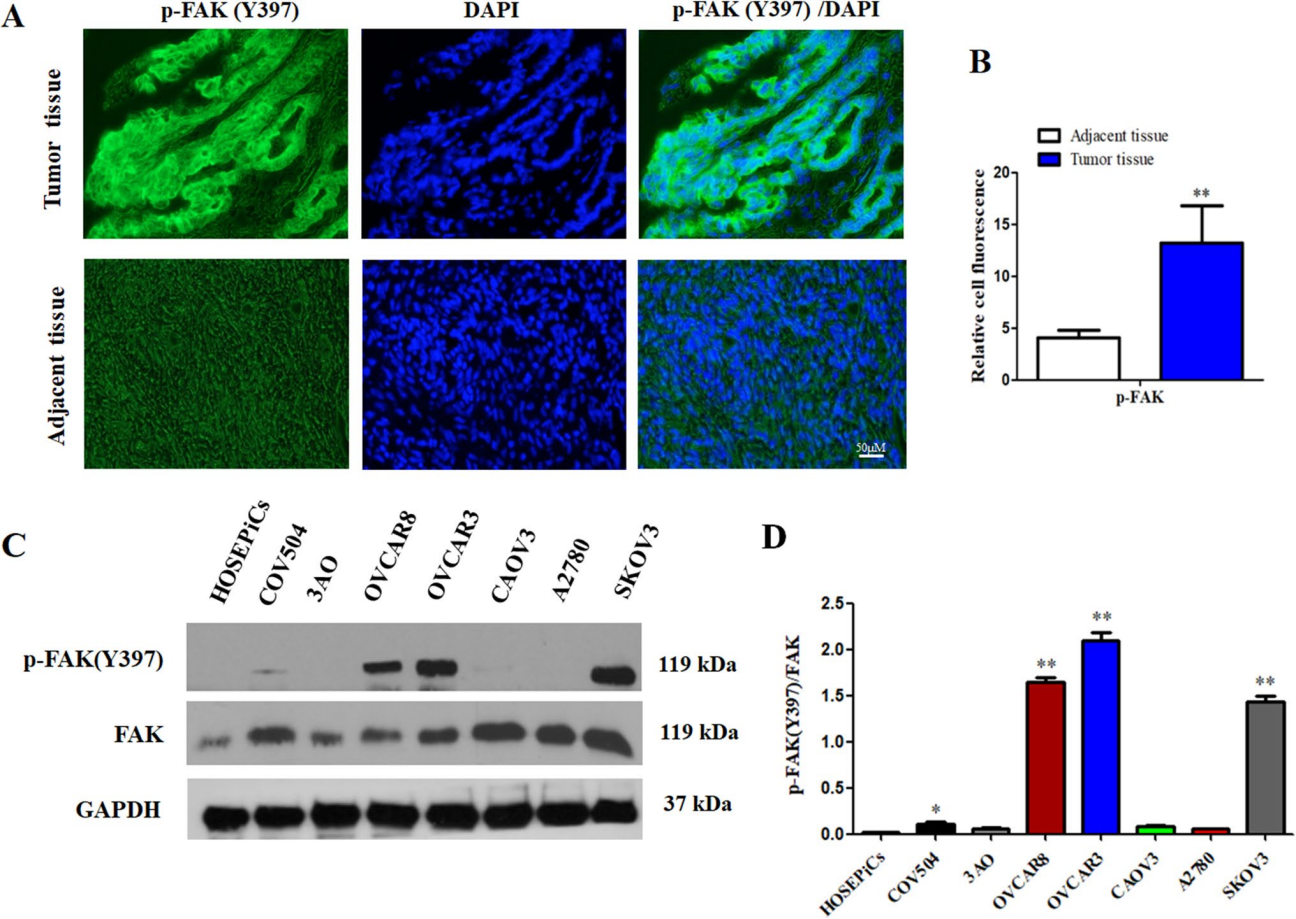
Phosphorylated focal adhesion kinase (p-FAK) (Y397) expression is upregulated in ovarian cancer tissue and cells. **A**, **B** p-FAK(Y397) expression in ovarian cancer tissue was upregulated than that in the adjacent non-tumor tissues. **C**, **D** p-FAK(Y397) protein expression was upregulated in SKOV3 and OVCAR3 cell lines as compared to HOSEpics cells. The data were presented as mean ± SD of three independent experiments. *P < 0.05, **P < 0.01

Firstly, we analyzed p-FAK expression in ovarian cancer tissues and cell lines. As a result, p-FAK expression in tumor tissue was upregulated than that in the adjacent non-tumor tissue (Fig. 1A, B). In contrast, SKOV3 and OVCAR3 cell lines exhibited higher protein expressions of p-FAK (Y397) than in HOSEpics cells (Fig. 1C, D). Therefore, we next assessed FAK inhibitor BI853520 in SKOV3 and OVCAR3 cells which demonstrated relatively high levels of p-FAK(Y397) activation in this study.

### BI853520 inhibits p-FAK in ovarian cancer cells

**Fig. 2:**
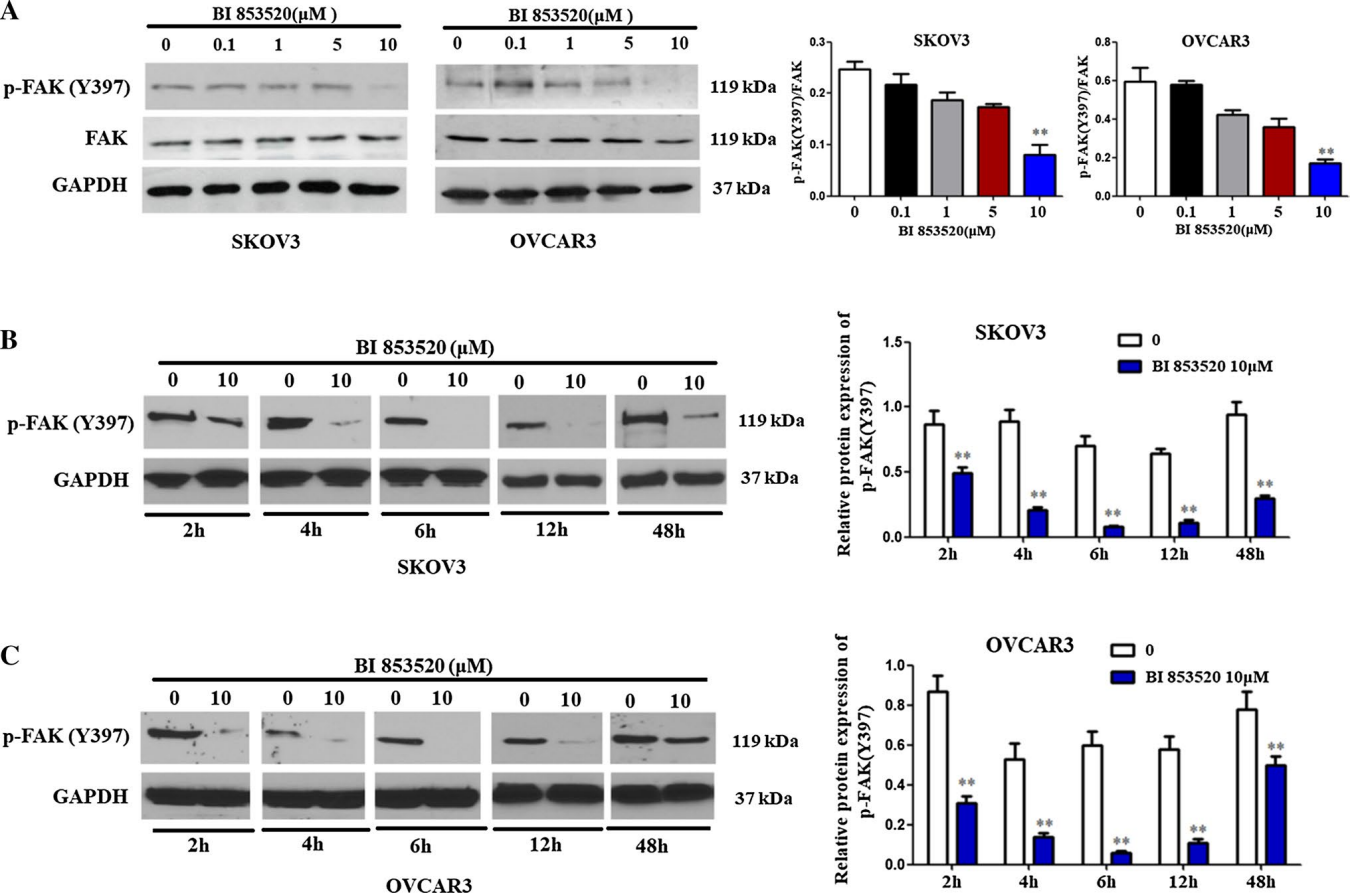
BI853520 inhibition of p-FAK (Y397) phosphorylation in ovarian cancer cells. **A** Protein expression levels of p-FAK (Y397)/FAK were measured by Western blot analysis in SKOV3 and OVCAR3 cells treated with BI853520 at different concentrations. **B**, **C** Protein expression levels of p-FAK (Y397) measured by Western blot analysis in SKOV3 and OVCAR3 ovarian cancer cells treated with BI853520 at 10 μM with different time points

The expressions of p-FAK following BI853520 treatments were detected to assess the pharmacologic effects of this highly selective inhibitor of FAK. Ovarian cancer cells were treated with varied doses of BI853520 (0.1–10 μM) for 12 h, then Y397-FAK phosphorylation was assessed using western blot. p-FAK expression ranged from less than 10% inhibition to more than 90% inhibition with an increasing doses of BI853520 treatments in both ovarian cancer cell lines (Fig. 2A). The ratio of phospho- to total-FAK decreased in both cells. This suggested that BI853520 resulted in a dose-dependent decrease of p-FAK(Y397) expression without effects on total FAK expressions in ovarian cancer cells.

In addition, ovarian cancer cells were treated following a range of time points with 10 μM BI853520 to determine its onset and duration. Decreased Y397-FAK caused by BI853520 treatment occurred within 2 h and was continually reduced at least for 48 h (Fig. 2B, C), suggesting a fast inhibition of FAK in ovarian cancer cells.

### BI853520 decreases cell proliferation in ovarian cancer cells

**Fig. 3:**
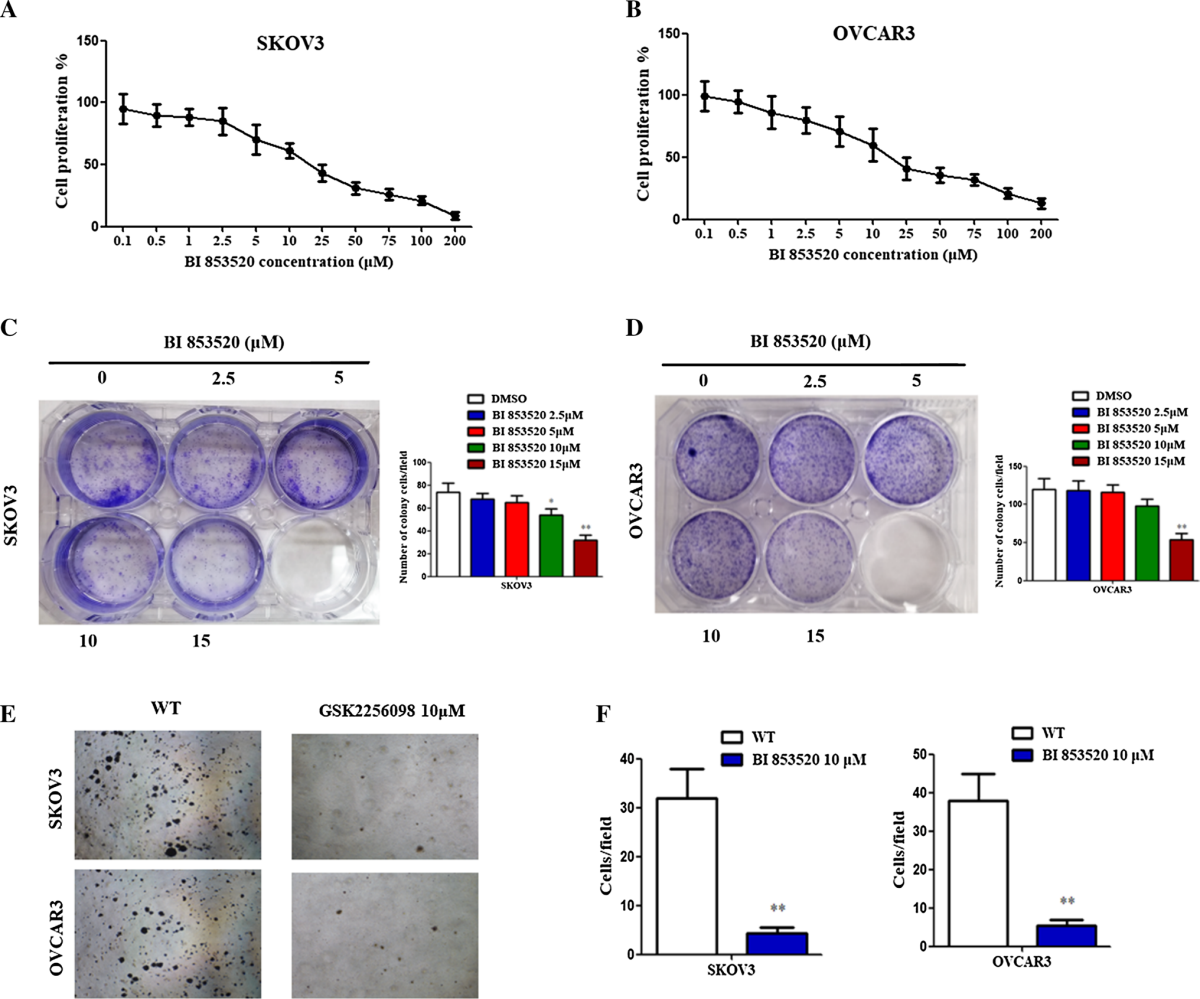
BI853520 inhibits cell proliferation and colony formation abilities in ovarian cancer cells. **A**, **B** Proliferation of SKOV3 and OVCAR3 cells was measured using MTT at different concentrations of BI853520 for 12 h. **C**, **D** Clonogenic ability of SKOV3 and OVCAR3 cells was measured using colony formation assay at different concentrations of BI853520 for 12 h. **E**, **F** Clonogenic ability of SKOV3 and OVCAR3 cells was measured using soft agar assay following treatment with BI853520 for 12 h. The data were presented as mean ± SD of three independent experiments. **P < 0.01

Cells were incubated for 12 h following various doses of BI853520 treatment (0, 0.1, 0.5, 1, 2.5, 5, 10, 25, 50, 75, 100, and 200 μM). Results showed that cell proliferation decreased with the increasing doses of BI853520 in both SKOV3 and OVCAR3 cell lines (Fig. 3A, B).

### BI853520 inhibits cell colony formation in ovarian cancer cells

Decreasing cell viability and proliferation can reduce the number of cells. To assess the effect of BI853520 on the cell clonogenic ability, colony formation assays were performed (Fig. 3C, D). Results showed no remarkable impacts on cell colony formation ability upon treatment with 5 μM BI853520 compared with the vehicle. However, at the concentration of 15 μM, decreased colony formations were observed in both cell lines. The dose-dependent decrease in colony formation indicated the inhibition effects of cell growth of BI853520.

Besides, to determine whether BI853520 attenuates anchoragein dependent growth, SKOV3 and OVCAR3 cells were cultured under agarose gel-suspended conditions (Fig. 3E, F). The colony cells in soft agar were significantly decreased when treated at a concentration of 10 μM of BI853520. These observations revealed that the inhibiting cell growth ability of BI853520 is associated with cell culture conditions.

### BI853520 attenuates cell migration and invasion in ovarian cancer cells

**Fig. 4:**
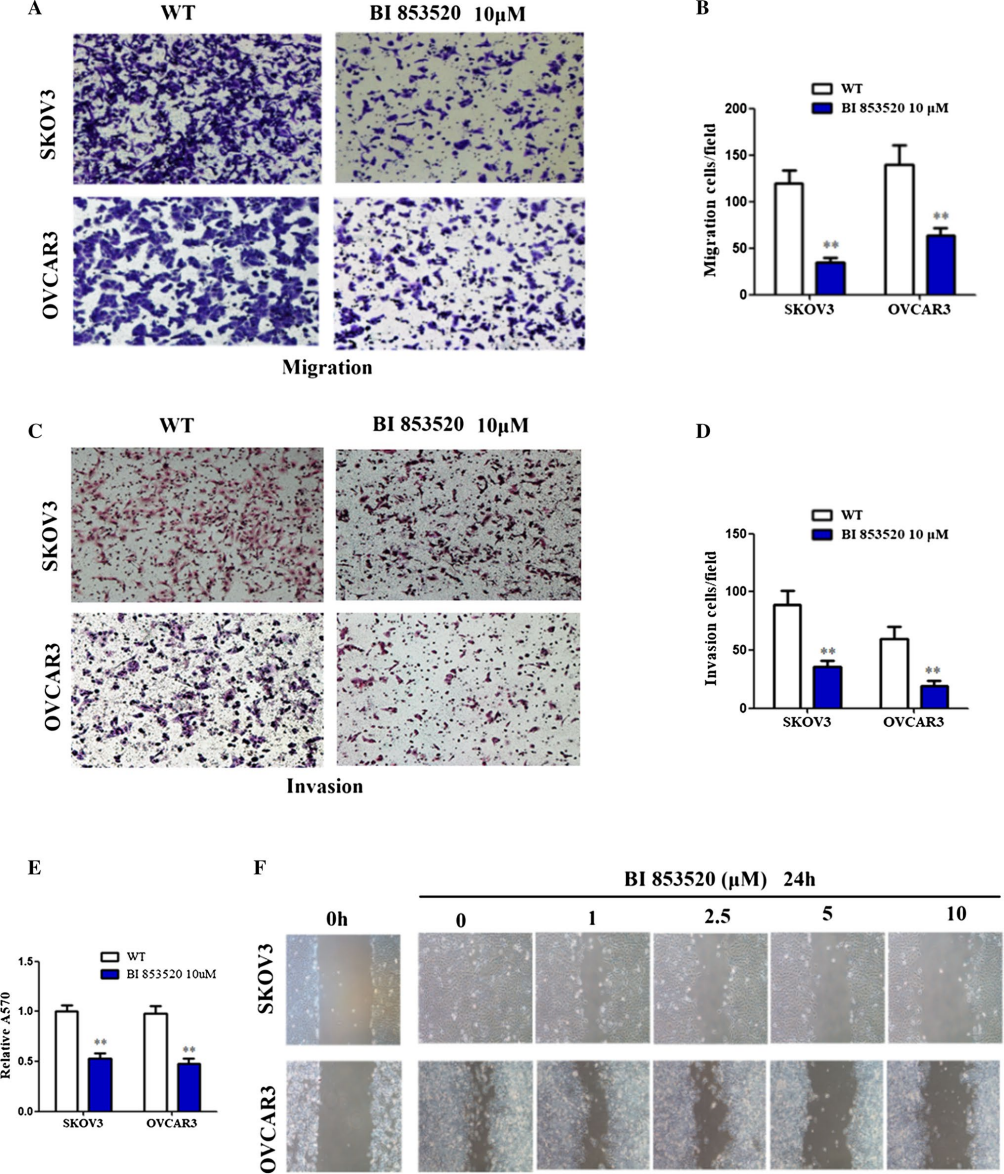
BI853520 attenuates cell migration and invasion in ovarian cancer cells. **A**, **B** The effect of BI853520 on cell migration in ovarian cancer cells was measured using transwell migration assays. **C**, **D** The effect of BI853520 on cell invasion in ovarian cancer cells was measured using transwell invasion assays. **E**, **F** Adhesion assay and wound healing assays were performed to detect cell adhesion and migration in SKOV3 and OVCAR3 cells. The data were presented as mean ± SD of three independent experiments. *P < 0.05, **P < 0.01

The migration cells were significantly decreased at a concentration of 10 μM of BI853520 in both cell lines (Fig. 4A, B). As we known, FAK play vital role in cell adhesion, therefore, adhesion assay and wound healing assay were performed to validate cell migration (Fig. 4E, F). Similarly, BI853520 impaired wound healing at a relatively low dose of BI853520 in both cells, while invasion ability was inhibited at a dose of 10 μM of BI853520 (Fig. 4C ,D). Our results suggested that BI853520 treatment can attenuate cell migration and invasion in ovarian cancer cells.

### BI853520 treatment induces anti-malignant phenotype in vitro

**Fig. 5:**
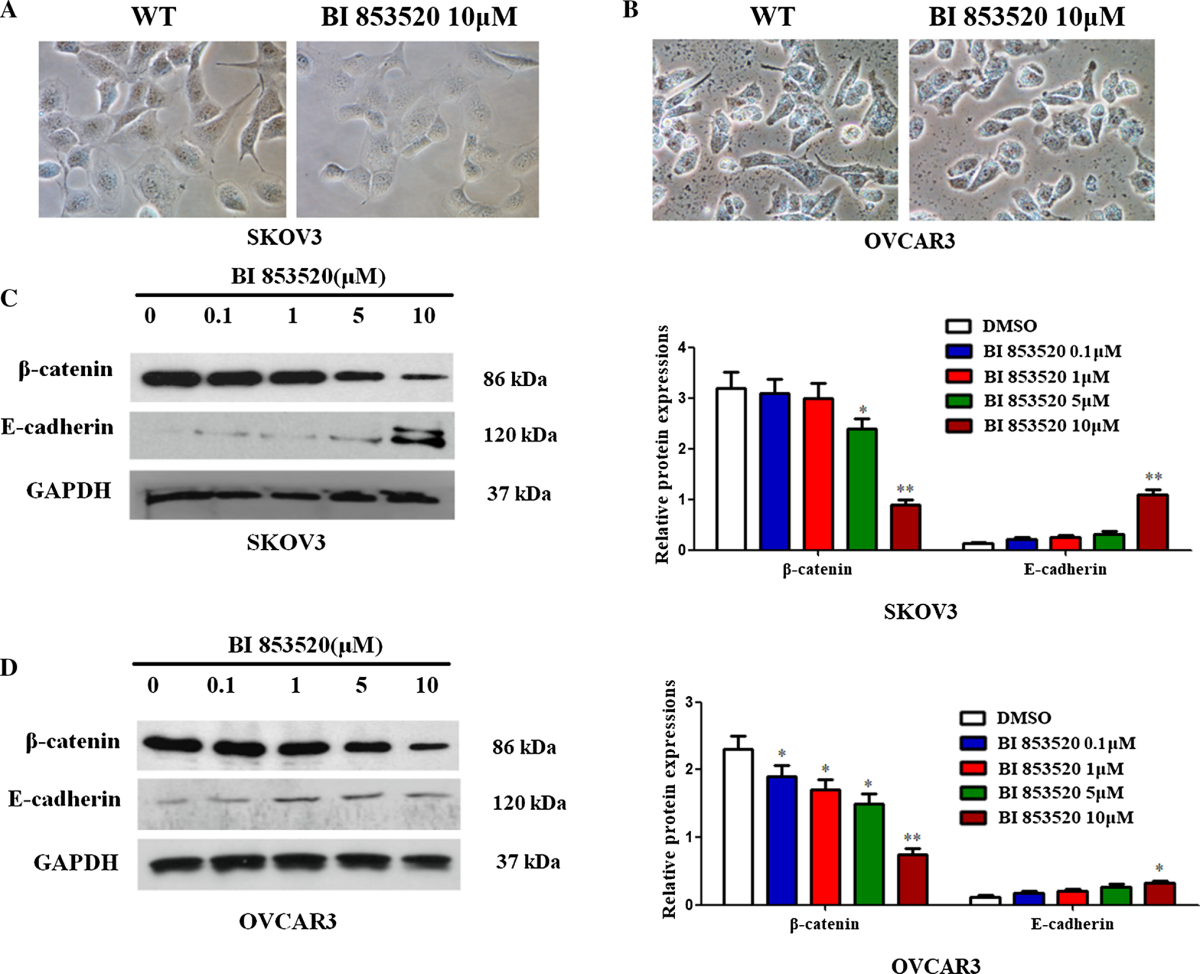
BI853520 inhibits EMT process in ovarian cancer cells. **A**, **B** Cell morphology with and without BI853520 treated SKOV3 and OVCAR3 cells for 24 h. **C**, **D** Protein expression levels of EMT marker genes were examined following treatment with BI853520 at different concentrations in both cell lines. The data were presented as mean ± SD of three independent experiments. *P < 0.05, **P < 0.01

Cells were incubated with 10 μM of BI853520 for 24 h. Results showed that BI853520 treatment with a dose of 10 μM altered the phenotype in both cell lines and cells morphologically resembled to epithelial phenotype status, which means that BI853520 treatment induces anti-malignant phenotype in vitro (Fig. 5A, B).

### BI853520 inhibits EMT process in ovarian cancer cells

The EMT associated genes were examined following treatment with different concentrations of BI853520 in both cell lines. As shown in Fig. 5, mesenchymal marker β-catenin decreased, while epithelial marker E-cadherin increased in BI853520 treated ovarian cancer cells, which showed that BI853520 inhibits EMT process in ovarian cancer cells (Fig. 5C, D).

### BI853520 increases biologic effects combined with chemotherapy

**Fig. 6:**
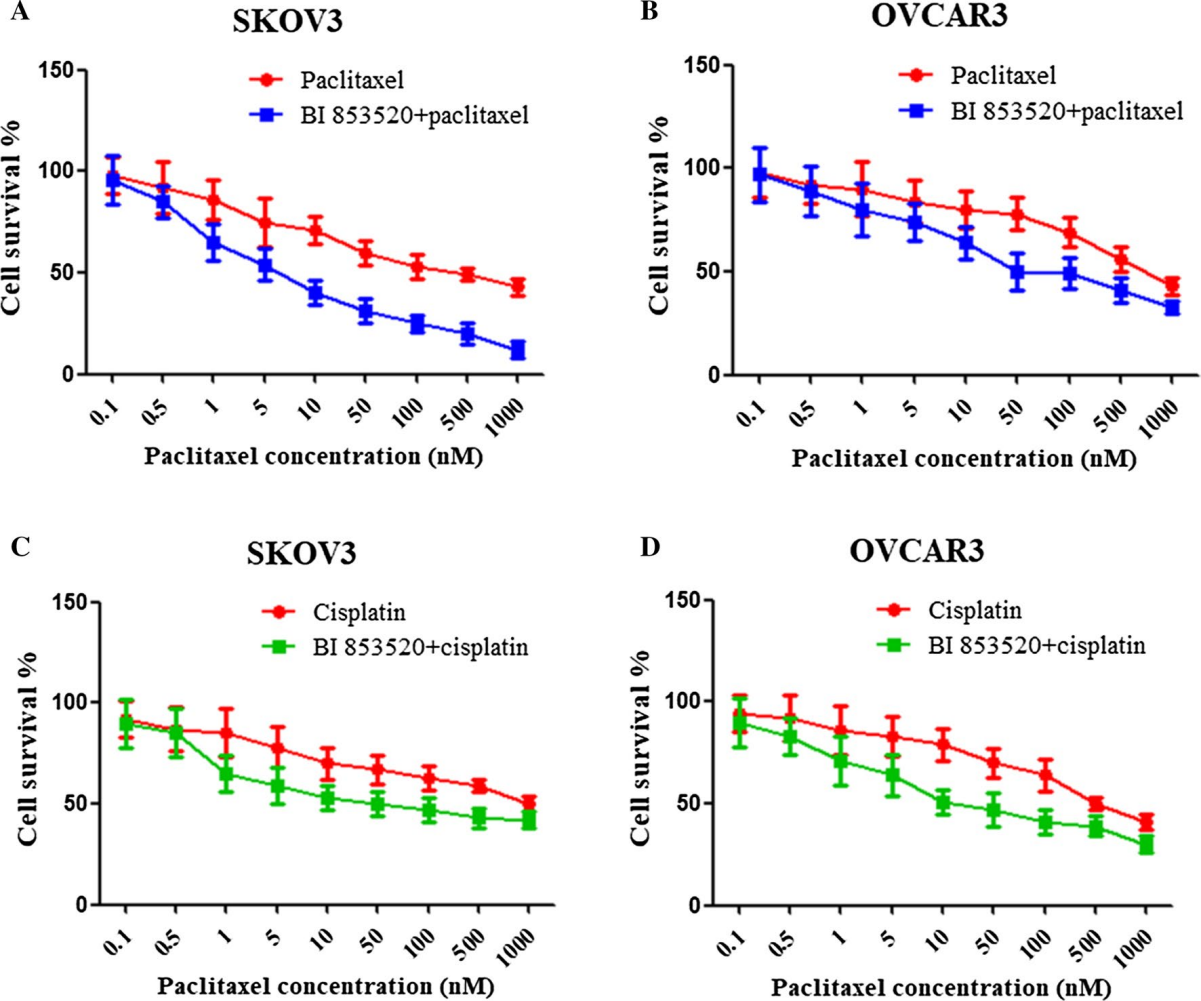
BI853520 increases biologic effects combined with chemotherapy. **A**, **B** Cell viability was assessed following treatment with 10 μM of BI853520 in combination with paclitaxel for 72 h with a range of concentrations. **C**, **D** Cell viability was assessed following treatment with 10 μM of BI853520 in combination with cisplatin for 96 h with a range of concentrations. The data were presented as mean ± SD of three independent experiments. **P < 0.01

Firstly, both cells were treated with chemotherapy drugs (paclitaxel or cisplatin) that used clinically for ovarian cancer treatment. Cell viability was assessed following combination treatment with BI853520 and paclitaxel for 72 h, or cisplatin for 96 h at various concentrations in SKOV3 and OVCAR3 cells (Fig. 6C, D). The results showed that BI853520 increased the sensitivity to clinical chemotherapy of ovarian cancer cells. Moreover, SKOV3 cells showed more sensitive than OVCAR3 cells when combined treatment with BI853520 and paclitaxel, while the response to treatment with BI853520 and cisplatin were similar in both cell lines, which suggested that BI853520 improves the sensitivity to chemotherapy of ovarian cancer cells.

### BI853520 inhibits PI3K/AKT/mTOR signaling pathway in ovarian cancer cells

**Fig. 7:**
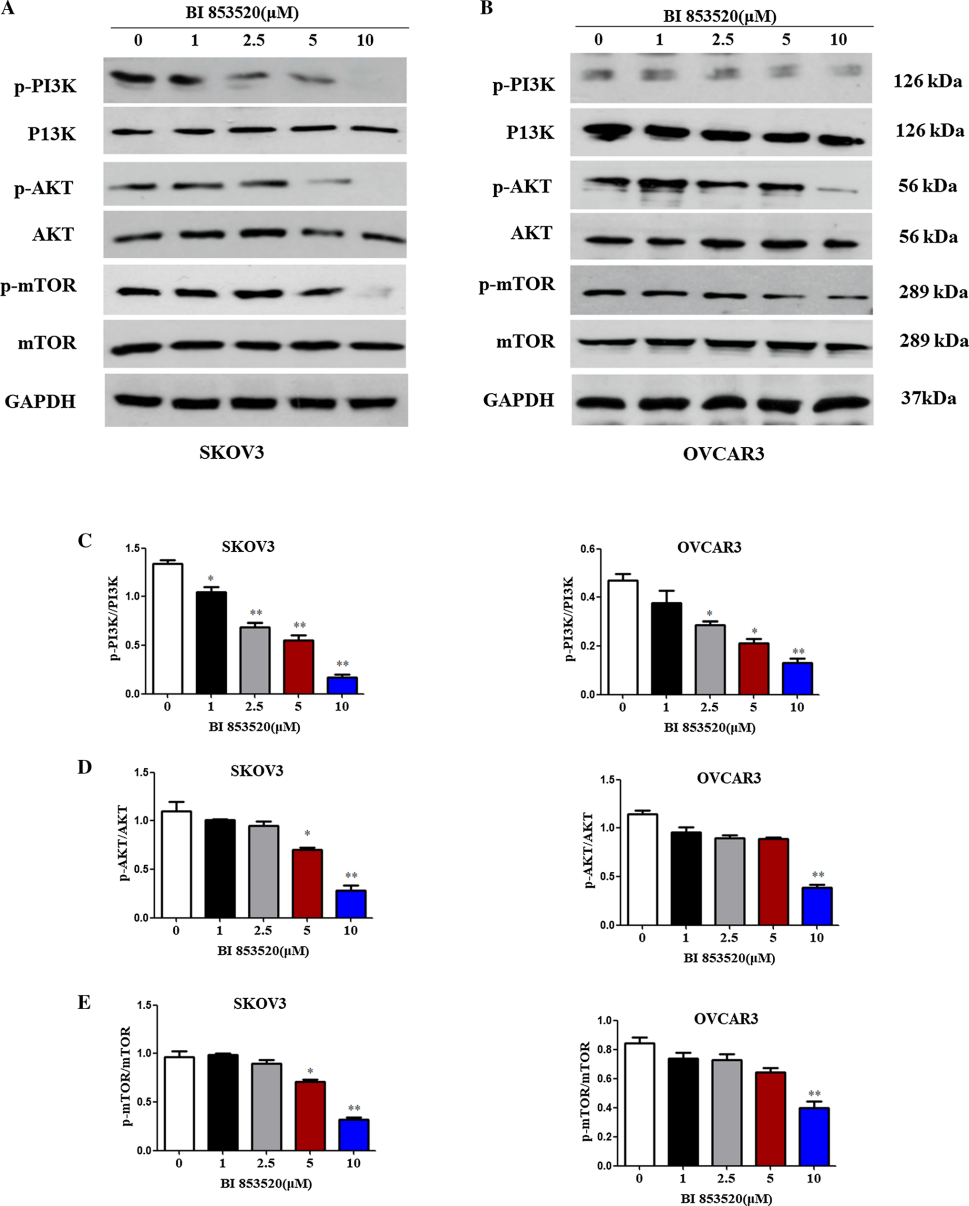
BI853520 inhibits PI3K/AKT/mTOR signaling pathway in ovarian cancer cells. **A**, **B** Protein expression levels of p-PI3K/PI3K, p-AKT/AKT and p-mTOR(S2448)/mTOR were measured by western blot analysis in SKOV3 and OVCAR3 cells treated with BI853520 at different concentrations. GAPDH was included as the loading control. **C**–**E**. The histograms of gray values of p-PI3K/PI3K, p-AKT/AKT and p-mTOR(S2448)/mTOR western blot results. The data were presented as mean ± SD of three independent experiments. **P < 0.01

As downstream genes of FAK signaling, the phosphorylation of PI3K/AKT/mTOR (p-PI3K/AKT/mTOR) indicates activation of PI3K/AKT/mTOR, which contributes to cell proliferation, survival and migration. The inhibition effects on p-PI3K/AKT/mTOR (S2448) were examined after incubating the cells with BI853520 for 12 h (Fig. 7A, B). Results showed that BI853520 treatment significantly decreased the protein expressions of p-PI3K/AKT/mTOR in both SKOV3 and OVCAR3 cells (lower p-PI3K/AKT/mTOR phosphorylation) (Fig. 7C–E). This observation demonstrated a pharmacological action of BI853520 in inhibiting PI3K/AKT/mTOR signaling pathway in ovarian cancer cells.

### BI853520 suppresses tumor growth in vivo

To determine the role of BI853520 in vivo, mouse models were performed. The mice were injected with SKOV3 cells treated with monotherapy or combination with chemotherapy. In the mouse model, tumor growth was inhibited in the BI853520 monotherapy group as compared to the normal control model. Besides, paclitaxel combined with BI853520 greatly reduced tumor growth of mice than that in the BI853520 or paclitaxel monotherapy group, which suggested that BI853520 improves the sensitivity to chemotherapy in vivo.

**Fig. 8:**
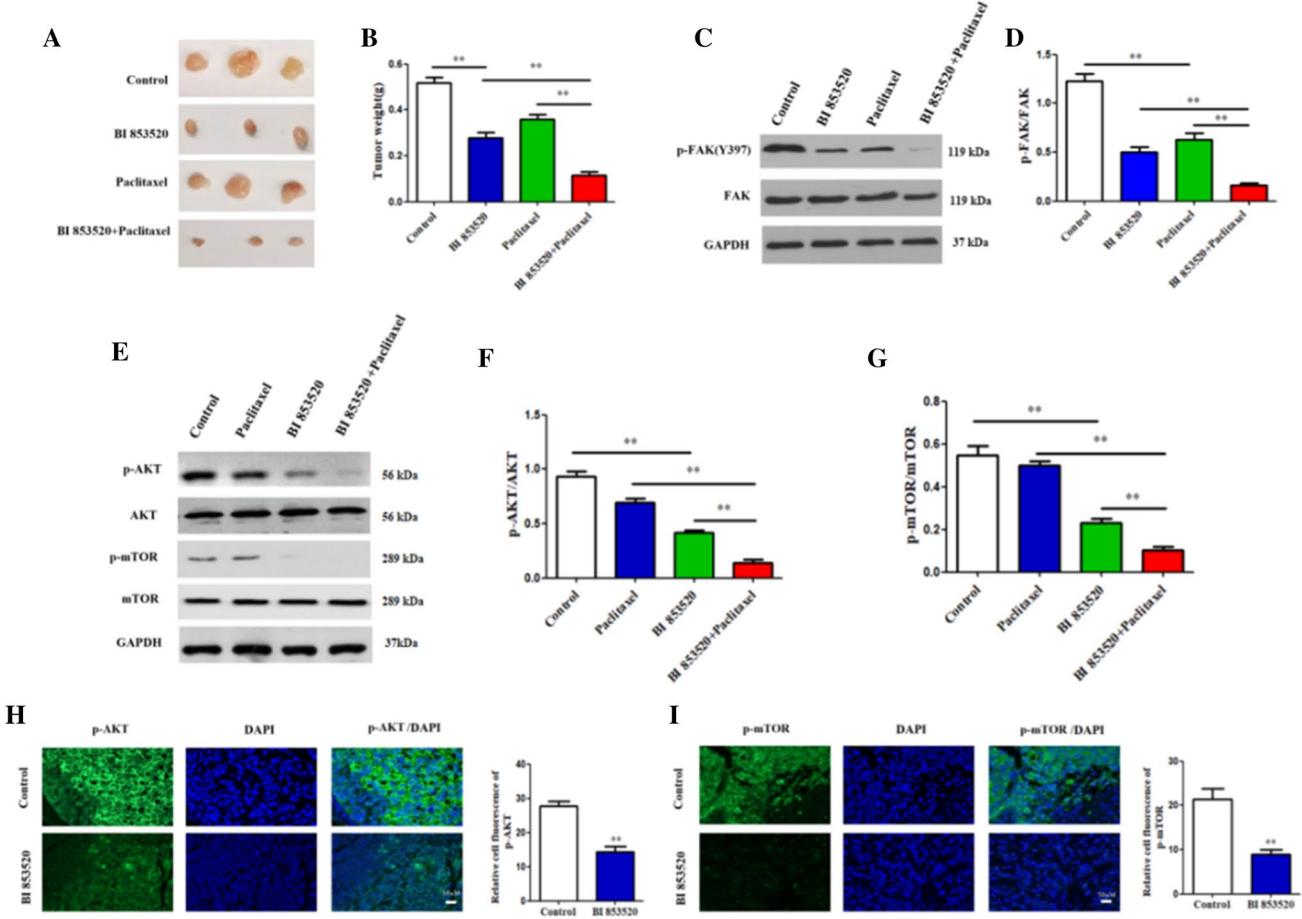
BI853520 attenuates orthotopic tumor growth through PI3K/AKT/mTOR signaling pathway in vivo. **A**, **B** The mean mouse weights following treatment with BI853520, paclitaxel monotherapy and combination treatment. **C**, **D** Protein expression levels of p-FAK (Y397) were examined in mice tumors following treatment with BI853520, paclitaxel monotherapy and combination treatment using Western blot analysis. **E**–**G** Protein expression levels of p-AKT/AKT and p-mTOR(S2448)/mTOR were measured in mice tumors following treatment with BI853520, paclitaxel monotherapy and combination treatment using Western blot analysis. **H**, **I** p-AKT and p-mTOR(S2448) in mice tumors were examined using imunofluorescence staining assay. The data were presented as mean ± SD of three independent experiments. **P < 0.01

Moreover, mouse weights in paclitaxel combined with BI853520 group were lower than those in the BI853520 or paclitaxel monotherapy groups (Fig. 8A, B). The results revealed BI853520 treatment could suppress ovarian tumor growth in vivo.

### BI853520 inhibits PI3K/AKT/mTOR signaling pathway in vivo

Western blot analysis further displayed that protein level of p-FAK(Y397) was decreased in mice tumors treated with BI853520. Moreover, a lower protein level of Y397-FAK phosphorylation was found in paclitaxel combined with BI853520 treatment group than in the BI853520 or paclitaxel monotherapy group (Fig. 8C, D), which is consistent with our results in vitro, that BI853520 improves the sensitivity to chemotherapy of ovarian cancer cells.

To detect whether BI853520 inhibits PI3K/AKT/mTOR signal pathway in vivo, AKT/mTOR (S2448) protein expressions were examined in mice tumors, as a result, mice tumors in BI853520 treatment group showed lower AKT/mTOR (S2448) protein expressions than that in control group. Besides, AKT/mTOR (S2448) protein expressions were further decreased in paclitaxel combined with BI853520 group than that in the BI853520 or paclitaxel monotherapy group (Fig. 8E–G). Furthermore, immunofluorescence staining assay was performed to detect activated p-AKT and p-mTOR in mice tumor slides, results showed that BI853520 treatment group had lower level of p-AKT and p-mTOR compared to normal control (Fig. 8H, I). Together, the data suggested BI853520 suppressed primary tumor proliferation in vivo through PI3K/AKT/mTOR signaling pathway.

## Discussion

FAK is a nonreceptor protein tyrosine kinase, and play a role in cell progression of several cancer cells [20]. FAK expression was reported associated with lymphatic metastasis and distant recurrence in ovarian cancer, uterine cancer and breast cancer [20, 21]. A number of FAK inhibitors have been taken under research as targeted therapies and proved to prevent tumor growth [22–24].

Several studies showed that overexpression of FAK led to an increase of cell proliferation of mammary tumor cells, whereas knockdown the expression of FAK decreased cell proliferation [25, 26]. In line with these findings, MTT results showed that BI853520 induced a reduction of cell proliferation in vitro. Besides, to further validate the anti-proliferative ability of BI853520 in vivo, animal models of ovarian cancer were performed. In the mouse model, tumor growth was inhibited in the BI853520 treatment group as compared to the normal control. Together, these data proved the anti-proliferative effect of BI853520 both in vitro and in vivo.

FAK is known a regulator in focal adhesion dynamics [27–30], therefore, the migratory activity was measured using wound healing assay in the presence of BI853520. BI853520 impaired wound healing ability even at 1 μM concentration of BI853520. Interestingly, similar results were proved by transwell migration assays, which revealed a strong migration inhibitory ability of BI853520. While in invasion assays, cells were inhibited only at a high dose of BI853520, which indicated the inhibition effect of BI853520 on cell invasion may be a result of its anti-proliferation effect.

Additionally, a recent study by Duangmani et al. on uterine cancer mouse models showed that GSK2256098, a FAK inhibitor enhanced the effect of chemotherapy drugs [29]. Halder et al. demonstrated that the combination treatment with TAE226, a FAK inhibitor and docetaxel led to a notable suppression in ovarian carcinoma [31]. In the current study, the effects of BI853520 in combination treatment with paclitaxel and cisplatin were examined in both cells, and the findings revealed that BI853520 improved antitumor activity in combination with traditional chemotherapeutics, which was supported by prior studies. To further examine the effect of BI853520, orthotopic mouse models of ovarian cancer cells were performed, the mice were injected with SKOV3 cells treated with monotherapy or combination with chemotherapy. In the mouse model, tumor growth was inhibited in the BI853520 monotherapy group as compared to the normal control model. Besides, paclitaxel combined with BI853520 greatly reduced tumor growth of mice than that in the BI853520 or paclitaxel monotherapy group, which suggested that BI853520 improves the sensitivity to chemotherapy in vivo.

When cultured in a 3D environment, the colony formation cells was inhibited at 10 μM of BI853520, while in sphere formation of 2-D colony formation, no remarkable impacts on cell colony formation ability upon treatment with 5 μM BI853520 compared with the vehicle. However, at the concentration of 15 μM, decreased colony formations were observed in both cell lines. These results indicated that inhibition of cell colony formation of BI853520 is functional effectively in specific cell culture conditions, which were in consistent with previous studies [32, 33].

E-cadherin, an EMT associated gene, was reported contributed to the sensitivity of FAK inhibitor mediated cell invasion and migration in malignant pleural mesothelioma cells [34]. In the current study, we detected EMT associated genes upon treatment with BI853520. The findings showed that mesenchymal marker β-catenin was downregulated, while the epithelial marker E-cadherin was upregulated in BI853520 treated SKOV3 and OVCAR3 cells compared to controls, which showed that BI853520 treatment inhibited EMT process in ovarian cancer cells. Besides, BI853520 treatment induced anti-malignant phenotype in vitro. This indicated that the EMT markers may serve as actionable biomarkers of BI853520 treatment in ovarian cancer cells.

To determine the underlying molecular mechanism of BI853520 in ovarian cancer cells, the activity of downstream PI3K/AKT/mTOR signaling pathway was examined. Results showed BI853520 decreased p-PI3K/AKT/mTOR phosphorylation in both cells. Besides, p-PI3K/AKT/mTOR phosphorylation in vivo tumor tissues were also decreased in BI853520 treatment group than that in the control group. Immunofluorescence staining results of mice tumor further proved that activated p-AKT and p-mTOR were decreased following BI853520 treatment compared to controls. In summary, the data suggested BI853520 suppressed primary tumor proliferation both in vitro and in vivo through PI3K/AKT/mTOR signaling pathway.

## Conclusion

Taken together, our research is the first to identify that focal adhesion kinase inhibitor BI853520 inhibits cell proliferation, migration and EMT process through PI3K/AKT/mTOR signaling pathway in ovarian cancer.

## Data Availability

All the data were included in this manuscript.
